# Glucosamine regulates macrophage function in heart failure

**DOI:** 10.1002/ctm2.819

**Published:** 2022-04-22

**Authors:** Marcin Wysoczynski, Jonah Stephan, Shizuka Uchida

**Affiliations:** ^1^ Diabetes and Obesity Center University of Louisville School of Medicine Louisville Kentucky USA; ^2^ Center for RNA Medicine Department of Clinical Medicine Aalborg University Copenhagen SV Denmark

**Keywords:** glucosamine, heart failure, macrophage, signaling

## 1 BACKGROUND

Healing myocardium after ischemic injury due to infarction requires sequential recruitment of immune cells to facilitate necrotic tissue clearance and subsequent activation of mesenchymal compartment to initiate deposition of extracellular matrix resulting in formation of a collagen‐based scar tissue. Although post‐myocardial infarction (MI) leukocytosis is associated with adverse outcomes, the last two decades of investigation revealed that immune cells actively participate in tissue repair and homeostasis after MI.^1^ Macrophages are the central component of the healing myocardium. Tissue‐resident macrophages deposited during embryonic development actively maintain tissue homeostasis, but also orchestrate immune cells infiltration after ischemic injury. In addition to tissue‐resident macrophages, circulating monocytes of bone marrow and splenic origin are rapidly recruited to the heart after MI.^2^ Following extravasation, monocytes differentiate to macrophages and facilitate necrotic tissue clearance via efferocytosis. Macrophage plasticity allows them to acquire various secretory specialised phenotype. In general, in response to the local tissue environment, monocyte‐derived macrophages can differentiate into classical pro‐inflammatory or alternative reparative and pro‐resolving phenotype.^2^ The general macrophage contributions to infarct healing have been established; however, mechanistic aspects involved in regulation of macrophage number and function remain cryptic.

Among numerous post‐translational protein modifications, there is emerging evidence that O‐linked β‐N‐acetylglucosamine protein modification has immunoregulatory functions.^3^ O‐linked β‐N‐acetylglucosaminylation (O‐GlcNAcylation) is a subtype of glycosalyation that involves addition of O‐GlcNAc onto serine and threonine residues of nuclear or cytoplasmic proteins by O‐GlcNAc transferase (OGT). O‐GlcNAcylation overlaps the sites of protein phosphorylation.^3^ This suggests that GlcNAcylation may antagonise protein actions mediated by phosphorylation. However, there is no consensus regarding the functional outcome of protein GlcNAcylation in macrophages. Several studies demonstrated that macrophage protein GlcNAcylation regulates various aspects of macrophage biology. To name a few, GlcNAcylation of STAT3 on threonine 717 (T717) antagonises phosphorylation and reduces IL‐10 expression in lipopolysaccharide (LPS)‐stimulated macrophages. By this means, it suppresses anti‐inflammatory actions of macrophages.^4^ In contrast, other studies show that hyper‐O‐GlcNAcylation correlates with anti‐inflammatory actions of macrophages in sepsis. Glucosamine (GlcN) stimulation induces hyper‐O‐GlcNAcylation of proteins and reduces transcriptional activity of NFκB signaling in macrophages upon LPS stimulation.^5^ Similarly, GlcN treatment has been associated with M2 macrophage polarization in an LPS‐induced septic lung injury via O‐GlcNAcylation of nucleocytoplasmic proteins.^6^ Together, these data strongly indicate that protein GlcNAcylation has context‐dependent effects on macrophage effector function in various systemic inflammatory diseases. However, the role of macrophage protein GlcNAcylation in pathophysiology of cardiovascular disease remains understudied.

## 2 COMMENTARY

In the recent issue of *“Clinical and Translational Medicine”*, Zhou^7^ and colleagues demonstrate that GlcN administration preserves ventricular structure and function in the mouse model of MI‐induced heart failure. Two treatment protocols were tested: early (starting 1 day before MI) and late (starting 3 days after MI). Each protocol consisted of six daily intraperitoneal administrations of GlcN (300 mg/kg/day). Ventricular function measured with echocardiography revealed that GlcN administration improved left ventricular ejection fraction (EF) and fractional shortening (FS) at 7, 14 and 28 days after MI in both early and late treatment groups. Histological analysis of the heart sections at 28 days after MI exhibited reduction of the scar function in the treatment groups compared to the vehicle control group. Immunoprofiling with flow cytometry shows that the GlcN treatment increased Ly6C^Low^ and decreased Ly6C^High^ macrophage contents. Additionally, histological analysis demonstrated accumulation of CD206^Pos^ macrophages but no change in total macrophage content in the hearts of mice treated with GlcN compared with vehicle controls. These data suggest that the GlcN treatment impacts skewing of macrophage towards reparative and pro‐resolving phenotype, and as a consequence facilitate myocardial repair after MI. Mechanistic studies revealed that the GlcN treatment increased protein O‐GlcNAcation in myeloid cells. More detailed analysis shows an increase in STAT1 O‐GlcNAcation, which results in increased expression of Cx3cr1. Thus, the authors concluded that GlcN administration facilitates myocardial recovery after ischemic injury via increase in recruitment of CX3CR1^Pos^ macrophages skewed towards a reparative and pro‐resolving phenotype.^7^

This study adds new knowledge to the existing literature regarding immunomodulatory effects of GlcN supplementation. In addition to the effects of GlcN administration on myocardial repair, the novel observation suggests that the classical proinflammatory actions of STAT1 in macrophages are antagonised by O‐GlcNAcation.^7^, ^8^, ^9^ That further support the hypothesis that protein O‐GlcNAcation may have antagonistic effect on protein phosphorylation. However, multitude of question remains. Although the authors focus on the role of GlcN supplementation on STAT1 signaling, there are numerous of other pathways that are affected by GlcN and protein O‐GlcNAcation.^10^ Thus, it remains inconclusive whether enhanced myocardial repair after GlcN treatment in fact is solely due to STAT1 O‐GlcNAcation or via other mechanisms. More mechanistic studies would be warranted to explore this mechanism. Furthermore, GlcN shows clear benefit when injected acutely during ongoing inflammatory phase and scar formation. Because accumulation of macrophages in the chronic phase after MI contributes to progressive scarring and exacerbates ventricular dysfunction, it would be imperative to test the impact of GlcN administration on chronic non‐resolving inflammation and remodelling in chronic models of heart failure.^2^ Nevertheless, the current study warrants large preclinical studies in large animals (e.g., pigs and nonhuman primates) and perhaps clinical studies in MI patients to evaluate the efficacy of GlcN supplementation on the outcomes of post‐MI heart failure.
